# Pathway-Based Personalized Analysis of Pan-Cancer Transcriptomic Data

**DOI:** 10.3390/biomedicines9111502

**Published:** 2021-10-20

**Authors:** Cong Pian, Mengyuan He, Yuanyuan Chen

**Affiliations:** Department of Mathematics, College of Science, Nanjing Agricultural University, Nanjing 210095, China; piancong@njau.edu.cn (C.P.); 2019111002@njau.edu.cn (M.H.)

**Keywords:** pan-cancer, pathway deregulation scores, cancer-specific deregulated pathways, prognostic pathways, prognostic model

## Abstract

The occurrence of cancer is closely related to the deregulation of certain pathways. Based on pathway deregulation scores (PDS) inferred by the Pathifier algorithm, we analyzed transcriptomic data of 13 different cancer types in The Cancer Genome Atlas database to identify cancer-specific deregulated pathways and prognostic pathways. The results showed that the individual-specific pathway deregulation scores can clearly distinguish different cancer types and their tumor-adjacent tissues. In addition, the cancer-specific deregulated pathways and prognostic pathways of different cancer types had high heterogeneity, and the identified cancer prognostic pathways have been reported to be closely related to the corresponding cancers. Furthermore, we also found that cancers with more deregulation pathways tend to be malignant and have worse prognoses. Finally, a Cox proportional Hazards model was constructed based on the prognostic pathways; this model successfully predicted survival and prognosis based on data from cancer samples. In addition, the performance of the breast cancer prognostic model was validated with an independent data set in the METABRIC database. Therefore, the prognostic pathways we identified have the potential to become targets for the treatment of cancer.

## 1. Introduction

The latest data released by the International Agency for Research on Cancer (IARC) of the World Health Organization show that, in 2020, there were 19.3 million new cancer cases diagnosed worldwide and nearly 10 million deaths from cancer [1]. Although there have been a large number of studies related to the prevention, diagnosis, and treatment of cancer, its complicated pathogenic mechanism is still unclear. With the continuous development of high-throughput technology, a large amount of omics data have been generated, which provides unprecedented opportunities for in-depth study of the mechanisms underlying the occurrence and development of cancer and of cancer prevention and treatment strategies.

Years of research have shown that cancer is generally considered to be driven by the continuous accumulation of somatic mutations throughout an individual’s life, as well as by changes in epigenetics and transcription. Genes do not exist in isolation but interact with each other to form an organic biological network. The network-based cancer prognostic prediction model is more robust than the prediction model based on a single gene. Deregulation of biological pathways or biological networks often leads to the occurrence and development of cancer. Therefore, mining cancer-specific deregulated pathways can better explain the mechanisms of cancer occurrence and development at the system level [2,3].

At present, there are many methods for performing pathway analysis by combining high-throughput data. However, almost all of these methods can only characterize the path activity of the entire sample set and cannot provide information about the individual-specific related deregulated pathways in a specific cancer sample. For example, Efroni S et al. objectively identified pathways associated with malignancy, stage, and outcome in cancer through application of an analytic approach that systematically evaluates differences in the activity and consistency of interactions within canonical biologic processes [4]. Emmert-Streib F et al. discussed one popular way of integrating biological knowledge into large-scale genome-wide measurements, namely, the identification of functionally related genes (pathways) enriched or differentially expressed in gene expression data [5].

In response to the high heterogeneity of cancer among individuals, Drier Y et al. introduced Pathifier, an algorithm that infers pathway deregulation scores (PDS) for each cancer sample on the basis of expression data [6]. The algorithm transforms gene-level information into pathway-level information, thereby quantifying the deregulation level of each sample in terms of each biological pathway. The approach is phenomenological and, unlike the method of Vaske et al. [7], requires neither knowledge of the inter-relations between thousands of “biomolecular entities” nor measurement of their status. Studies have shown that (for glioblastoma and colon cancer), Pathifier can find the relevant pathways for cancer individuals [6]. In addition, the PDS score of the pathway can also successfully distinguish the subtypes of breast cancer, and the prognostic model based on the PDS score can be utilized with accommodated crosstalk to identify disease-specific features in order to predict prognosis from samples of hepatocellular carcinoma (HCC) [8,9,10].

Based on the Pathifier algorithm and its inferred PDS, we analyzed 13 different types of cancer transcriptomic data and clinical data in The Cancer Genome Atlas (TCGA) database to identify cancer-related deregulated pathways and further identify cancer prognostic pathway. The results showed that the individual-specific PDS can clearly distinguish different cancer types and their tumor-adjacent tissues. In addition, the deregulated pathways and prognostic pathways of different cancer types have high heterogeneity, and the identified cancer prognostic pathways have been reported to be closely related to the corresponding cancers. Finally, a Cox-proportional Hazards (Cox-PH) model based on the cancer prognostic pathways was constructed. In addition, we used the expression data of breast cancer in METABRIC to validate the performance of the prognostic model for breast cancer. The results showed that the model also predicted the prognosis of breast cancer well in the independent validation set.

We proposed a pathway-based cancer prognostic prediction model based on the Pathifier method. Through this model, individualized pathway risk scores were inferred for the pathway deregulation in 13 cancer types in the TCGA database, and pathways related to cancer prognosis were identified based on the PDS. The results showed that the PDS can distinguish different cancer types well and that there are significant differences in the prognostic pathways and cancer-specific deregulation pathways.

In this study, we used the Pathifier algorithm to calculate the PDS of a single sample rather than an aggregate group of samples. This method converts gene-level information into pathway-level information at the individual level, allowing the characterization of a single sample in a biological system. The PDS was used to identify cancer-specific deregulated pathways and prognostic pathways. Then, we constructed a pathway-pathway association network to explore the relationships among the prognostic pathways. Finally, Cox-PH models were constructed based on the prognostic pathways. These models can predict the survival states well in different cancer types respectively. In addition, the prognostic model for breast cancer was validated with an independent data in the METABRIC database. Thus, we believe that our pathway-based models are reliable for prognostic prediction based on pan-cancer data. In addition, this work may improve the development of precision medicine.

## 2. Materials and Methods

### 2.1. Data

RNA-seq data and clinical data for 13 cancer types, including 6140 cancer samples and 651 tumor-adjacent tissues, were downloaded from TCGA database (http://tcga-data.nci.nih.gov/tcga/ accessed on 24 June 2020) [11] by using TCGA-Assembler 2 (ver 2.0.6, http://www.compgenome.org/TCGA-Assembler/). Additional clinical data of all samples were downloaded using the R package RTCGA (version 1.22.0). RNA-seq data and clinical data for 1904 breast cancer samples were downloaded from the METABRIC database (http://www.cbioportal.org/study/summary?id=brca_metabric accessed on 1 April 2021) [12] for validation, and these data were used as validation data. The number of the samples for each cancer type can be seen in Table 1.

A total of 185 KEGG pathways were downloaded from the MSigDB database (http://www.gsea-msigdb.org/gsea/msigdb/, accessed on 2 March 2020) [13].

### 2.2. Overview of the Approach

**Figure 1:**
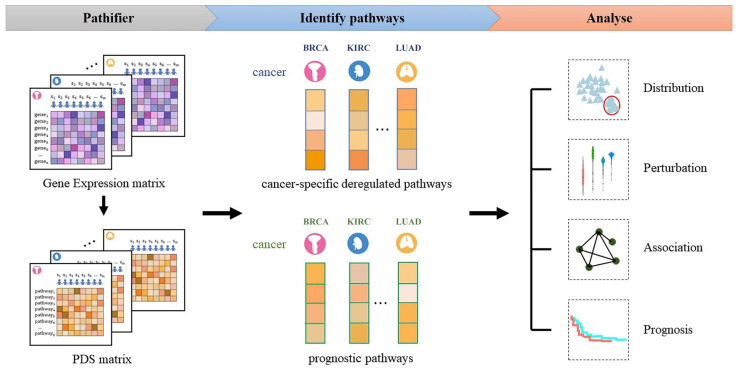
Flowchart of the approach.

There are three major steps in our method (see the flowchart in Figure 1). Step 1: Transform the gene expression matrix into the PDS matrix by using the Pathifier algorithm for each cancer type. Step 2: Identify cancer-specific deregulated pathways based on statistic model and prognostic pathways for each cancer type based on a Cox-PH model. Step 3: Analyze the distribution of the PDS in different cancers, identify the deregulated pathways in the cancer sample and all cancer samples with deregulation of this pathway, analyze the correlations among the prognostic pathways, and build prognostic prediction models for different cancer types based on the prognostic pathways.

### 2.3. Calculating the PDS

For any given pathway, Pathifier calculates a PDS for each cancer sample based on gene expression data [6]. The score represents the extent to which the activity of the pathway in a particular cancer tissue differs from that in normal cells of the same tissue.

Pathifier first calculates a score $D_{P}\left(s\right)$, which measures the extent to which the behavior of pathway P in sample s deviates from that in normal tissue. To determine the pathway deregulation score (PDS) of this pathway, the expression level of the $d_{P}$ gene belonging to pathway P  is used. Each sample s is a point in the $d_{P}$ dimensional space, and the entire sample set forms a point cloud. A (nonlinear) “principal curve” [14] is calculated to capture the variation of this cloud. Then, each sample is projected onto the curve, and the PDS is defined as the distance $D_{P}\left(s\right)$ measured along the curve between the projection of the sample s and the projection of the normal sample [6].

Based on the above process, the PDS of each sample in each pathway can be calculated. For each pathway, we calculate the mean and standard deviation of the PDS of all samples of each cancer type. If the mean PDS of a cancer sample $\bar{X_{i}^{c}}\left(i=1,\;2,\cdots ,13,\mathrm{where}\;i\;\mathrm{is}\;\mathrm{one}\;\mathrm{of}\;13\;\mathrm{cancer}\;\mathrm{types}\right)$ differed from the PDS mean $\bar{X_{i}^{n}}$ of a normal sample by two or more standard deviations $s_{i},$ that is,

(1) $$\left|\;\bar{X_{i}^{c}}-\bar{X_{i}^{n}}\right|\geq 2s_{i}$$

then this pathway is deregulated in the cancer sample; that is, the deregulated activity of this pathway in the cancer sample is significantly different from its activity in the normal sample. In this case, this pathway is defined as a deregulated pathway in the cancer sample, and the cancer sample is also called deregulated in this pathway.

### 2.4. Constructing Classifier to Distinguish Cancers from Normal

For better quantifying the differences between cancer samples and their tumor-adjacent tissue, we constructed random forest classifiers. Specifically, for each cancer type, we randomly selected 70% of all samples to train the random forest module, and tested the remaining 30% of samples. The performance ability was evaluated by the sensitivity (SN), specificity (SP), and accuracy (ACC), given by

$$\begin{array}{c} Sn=TP/TP+FN\\ Sp=TN/\left(TN+FP\right)\\ Acc=TP+TN/TP+FN+TN+FP \end{array}$$

where TP is True Positive, FP is False Positive, TN is True Negative, and FN is False Negative. This process was performed 100 times, and the mean values of Sn, SP, and ACC were calculated finally.

### 2.5. Identifying Deregulated Cancer-Specific Deregulated Pathways

Based on the PDS scores, we use the R package “heatmap” (version 1.0.12) to perform unsupervised hierarchical cluster analysis on all cancer samples by a statistical model. For a certain pathway, the mean PDS of all cancer samples $\bar{Y^{c}}$ and the mean PDS of all cancer samples of a certain cancer, $\bar{Y_{i}^{c}},i=1,\;2,\cdots ,13$, is calculated. If

(2) $$\left|\bar{Y_{i}^{c}}-\bar{Y^{c}}\right|\geq 2s$$

then the cancer type is deregulated in this pathway, which is called a cancer-specific deregulated pathway, where s represents the standard deviation of the mean PDS $\bar{Y^{c}}$ of this pathway in the 13 cancer types, that is,

(3) $$s=\sqrt{\frac{1}{13}\overset{13}{\underset{i=1}{\sum }}{\left(\bar{Y_{i}^{c}}-\bar{Y^{c}}\right)}^{2}}$$

### 2.6. Identifying Prognostic Pathways

According to the clinical data corresponding to the cancer samples, the survival outcomes (survival time and survival status) of the patients corresponding to the cancer samples were used as dependent variables, and a univariate Cox-PH model was established based on the PDSs. Pathways with *p* values less than 0.05 were identified as prognostic pathways.

Furthermore, a multifactorial Cox-PH model [15] for each cancer type was established based on the PDSs of the cancer prognostic pathways. According to the median risk score, the samples were divided into high-risk groups and low-risk groups, and Kaplan-Meier curves were generated. The “survival” (version 3.2-13) and “survminer” (version 0.4.9) packages in R/Bioconductor were used in the prognostic analysis.

In addition, gene expression data of breast cancer samples from the METABRIC database were used to verify the prognostic model for breast cancer, and the corresponding gene expression data of normal samples were also obtained from data for the 113 breast tumor-adjacent tissues in TCGA. Prognostic analysis was conducted after data standardization (min-max normalization) of the two databases.

For the identified prognostic pathways, the frequencies of the cancer prognostic pathways and their related pathways were determined based on the related pathway information in the KEGG database. The pathways with higher frequencies were used to construct a pathway-pathway association network to explore the relationships among the prognostic pathways.

## 3. Results

### 3.1. The Heterogeneity of the PDS

For tissues from 13 cancer types and the corresponding tumor-adjacent tissues in TCGA, the PDS score of each pathway in KEGG was calculated by the Pathifier algorithm.

**Figure 2:**
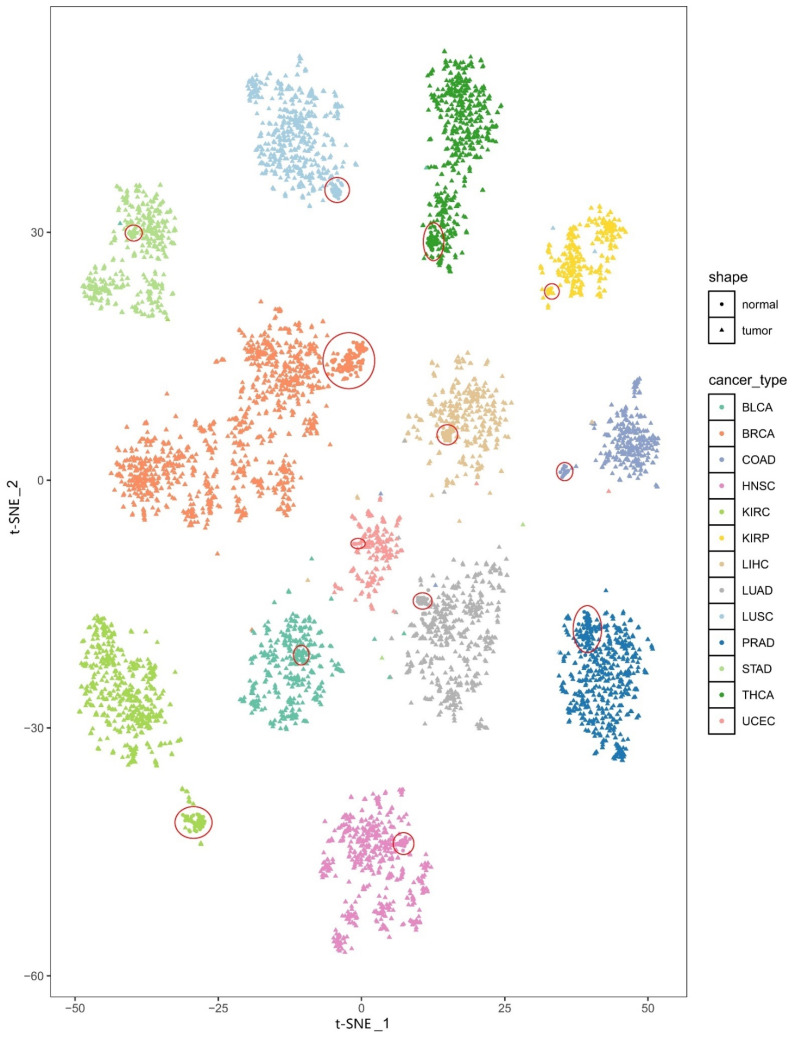
The t-SNE plot of the PDSs for all samples. Different colors represent different types of cancer. Triangles and dots are used to distinguish tissues of cancer types and the corresponding tumor-adjacent tissues, respectively. The red circle indicates the enrichment area of the tumor-adjacent tissues.

The t-SNE plot for the tissues from the 13 cancer types and the corresponding tumor-adjacent tissues is shown in Figure 2. We can easily see that samples of the same cancer type are clearly clustered together and that different cancer types are separated from each other well, indicating the high heterogeneity of the PDS across cancer types. In addition, we found that there is a good distinction between the tissue samples of different cancer types and the corresponding tumor-adjacent tissues and that the tumor-adjacent tissues also cluster together (the tumor-adjacent tissues are circled in red in Figure 2).

We constructed random forest classifier to distinguish cancer samples from their tumor-adjacent tissues. We can see that the performance of the random forest classifier is excellent to identify the cancer samples from their tumor-adjacent tissues. This result also shows that there is huge distance between cancer and adjacent tissue in terms of PDSs (see Table 2).

In addition, we found that cancer-specific deregulated pathways are related to the corresponding cancer. For example, the genes in MAPK signaling pathway, a COAD-specific pathway, encode a *MAPKKK* (*Raf*) and a *MAPKK* (*MEK1/2*), which are frequently mutated in colon cancer [16]. The JAK/STAT signaling pathway is identified as both a STAD-specific and THCA-specific pathway. The JAK/STAT signaling pathway has been shown to be aberrantly activated in thyroid cancer. In addition, the role of deregulated JAK/STAT signaling in the molecular pathogenesis of gastric cancer has been shown [17,18]. The Wnt signaling pathway, a COAD-specific pathway, is significantly deregulated in COAD. Studies have shown that mutations and defects in the Wnt signaling pathway are often found in colon cancer. In addition, the Wnt signaling pathway is constitutively deactivated by the destruction complex, which is assembled around the tumor suppressors APC and Axin and targets β-catenin for destruction [19].

**Figure 5:**
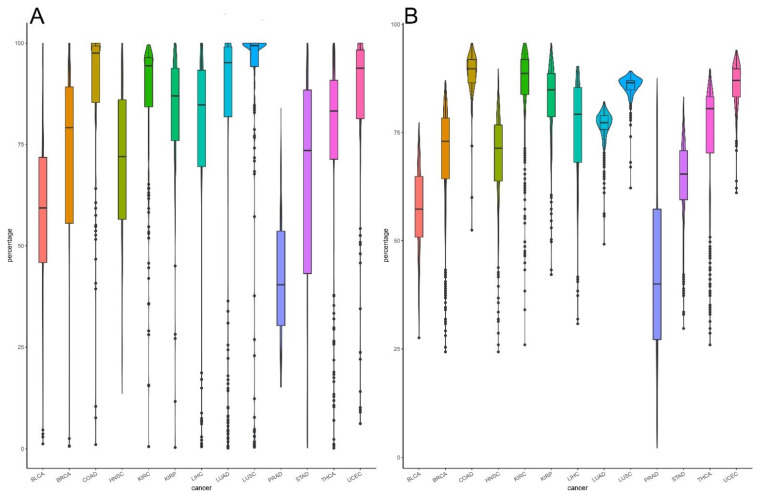
Violin plot showing deregulation in each cancer type: (**A**) the percentage of samples deregulated in each pathway in the 13 cancer types. The different colors represent different patient samples of each cancer type. COAD, LUSC, and KIRC showed deregulation of 89%, 86%, and 86% of pathways, respectively; (**B**) percentage of deregulated pathways in each patient. High percentages were observed in patients with COAD, LUSC, and KIRC.

In addition, we observed the heterogeneity of different cancers by analyzing the distribution of the proportion of cancer samples deregulated in each pathway. As shown in Figure 5A, among the 13 cancer types, there is a significant difference in the percentage of cancer samples deregulated in each pathway. COAD, LUSC, and KIRC have relatively high percentages of deregulated cancer samples, with averages of approximately 89%, 86%, and 86%, respectively. The percentage of UCEC samples (85%) is also very high. In contrast, PRAD has the lowest percentage (42%).

Similarly, the distribution of the deregulated pathways in each cancer sample is also significantly different across cancer types (Figure 5B). COAD, LUSC, KIRC, and UCEC have greater deregulation than other cancer types, and PRAD has significantly lower deregulation than other cancer types. This pattern is consistent with the results of related studies showing that COAD has the third highest incidence but second highest mortality [1]. In contrast, due to its slow growth, PRAD causes less damage to the human body and less distant metastasis, and most prostate cancers never cause symptoms or death [20,21].

### 3.2. Prognostic Pathways

Prognostic pathways for each cancer type were identified by univariate Cox-PH regression analysis. There were significant differences in the number of prognostic pathways among the different types of cancer. There were more prognostic pathways in KIRC, THCA, STAD, and HNSC and fewer in LUAD, PRAD, and UCEC. For example, 60 and 7 prognostic pathways were identified in KIRC and in PRAD, respectively, which were the highest and lowest numbers (see Supplementary Table S2). This difference may arise because the clinical outcomes of different cancer types are very diverse.

In addition, different cancer prognostic pathways rarely overlap. Thus, prognostic pathways have strong cancer specificity, as confirmed by Uhlen et al. [22]. Here, the prognostic pathways in the 13 cancers are distributed among different types, including pathways related to metabolism, organismal systems, environmental information processing, genetic information processing, cellular processes, and human diseases (see Supplementary Table S3).

Among the prognostic pathways and their related pathways, the MAPK signaling pathway, apoptosis, glycolysis/gluconeogenesis, PI3K-Akt signaling pathway, and cell cycle pathway have higher frequencies in the 13 cancer types. In addition, other known carcinogenic pathways, such as the p53 signaling pathway, Wnt signaling pathway, and TGF-beta signaling pathway, also show high connectivity. In particular, the MAPK signaling pathway shows the highest connectivity among these prognostic pathways and is related to the prognostic pathways in all cancers. In addition, the MAPK signaling pathway promotes cell survival by a dual mechanism comprising the post-translational modification and inactivation of a component of the cell death machinery and the increased transcription of pro-survival genes [23].

**Figure 6:**
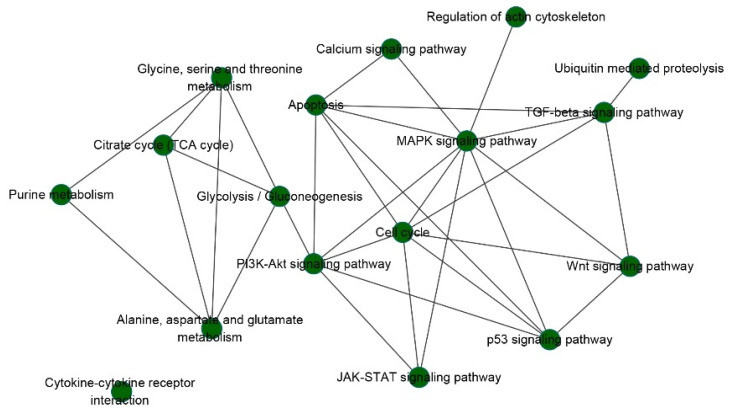
Pathway-pathway association network for the prognosis pathways and their related pathways.

Based on the assumption that similar diseases may be caused by deregulation of common oncogenic pathways, we constructed a cancer–cancer association network and found that most cancer types have very few shared prognostic pathways. In other words, most prognostic pathways are cancer specific. However, KIRC and KIRP shared the most common pathways, which indicates that cancer types with similar origin cell types share more oncogenic pathways (Table 3). In addition, a pathway-pathway association network for the prognostic pathways and their related pathways was constructed (Figure 6). MAPK signaling pathways and apoptosis, cell cycle, PI3K-Akt, TGF-beta, Wnt, Jak-STAT, and p53 signaling pathways were tightly connected, indicating that synergistic deregulation of these oncogenic pathways may contribute to tumorigenesis. However, we found that cytokine–cytokine receptor interaction with higher frequencies has less contact with other pathways, which may also be an important pathway. There is not enough relevant research on this pathway, so further exploration and study are needed.

In COAD, Notch signaling pathway was identified as prognostic pathway, which is consistent with the studies showing that the misregulation or loss of Notch signaling underlies a wide range of human disorders, from developmental syndromes to adult-onset diseases and cancer [24]. As an identified prognostic pathway, Toll like receptor signaling pathway is supported by a recent study showing that it is a potential therapeutic target in COAD, and correlated with COAD prognosis [25]. In addition, Toll like receptor signaling is involved in activating innate and adaptive immune responses and plays a critical role in COAD [25]. Elsewhere, we identified Cell cycle pathway as a prognostic pathway for HNSC. Cell cycle regulators are considered attractive targets in cancer therapy, and over expression of several of these cell cycle proteins induces or contributes to tumorigenesis, revealing their prominent oncogenic roles [26]. VEGF signaling pathway was identified as a prognostic pathway in HNSC. VEGF inhibitors play an increasingly important role in the management of solid tumors, and anti-VEGF therapy has established itself as one of the most important classes of drugs for the treatment of human cancer [27]. VEGF correlates with worse prognosis or outcome in general [28].

Moreover, N-Glycan biosynthesis, amino sugar, nucleotide sugar metabolism, and so on were prognostic pathways in BRCA; steroid hormone biosynthesis, insulin signaling pathway, and so on were prognostic pathways in KIRC; and other prognostic pathways among the different types of cancer may be specific pathways of different cancers. Our research indicates that they may be repurposed for the treatment of these cancers.

### 3.3. Prognostic Models Based on Pathways

**Figure 7:**
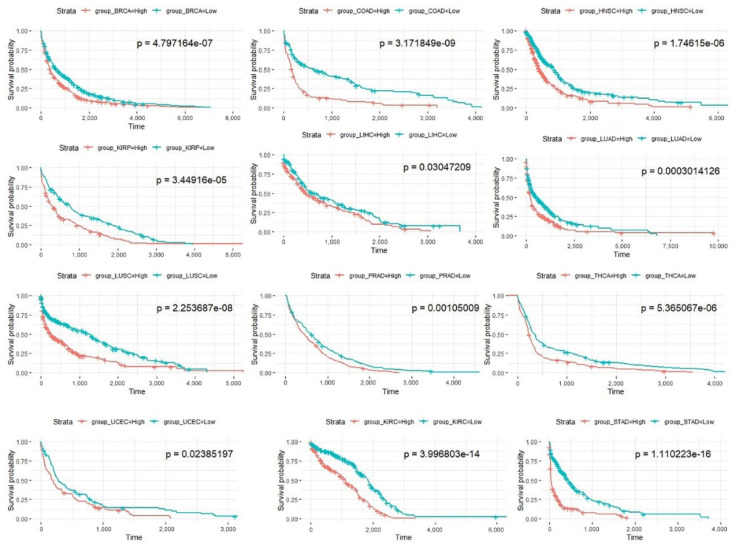
Kaplan-Meier curves of patients in the two risk groups dichotomized by deregulation of the prognosis pathways for 12 cancer types in TCGA. The *x* axis shows survival in days. The *y* axis shows the overall survival rate.

The Kaplan-Meier curves for all cancers are shown in Figure 7. It can be seen from the figures that the prognoses of patients in the high-risk score group are significantly less favorable (*p* < 0.05) than those of patients in the low-risk score group in 12 cancer types (except BLCA) in TCGA; this finding verifies the effectiveness of the prognostic model based on prognostic pathways.

**Figure 8:**
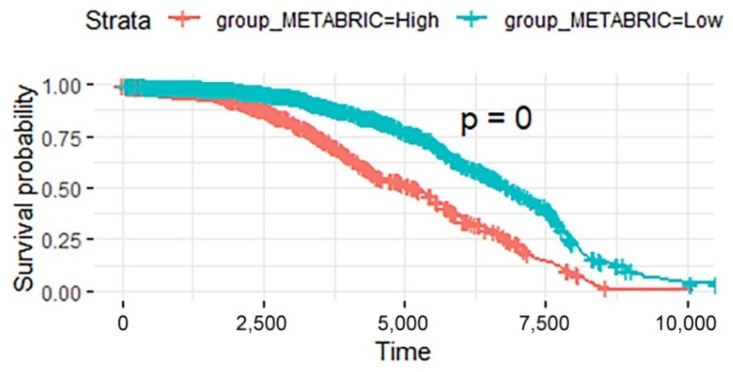
Kaplan-Meier curves of patients in the two risk groups dichotomized by deregulation of the prognosis pathways in BRCA data in METABRIC. The *x* axis shows survival in days. The *y* axis shows the overall survival rate.

In addition, the validation data in the METABRIC database were analyzed using the same process. The breast cancer samples in METABRIC were divided into a high-risk score group and a low-risk score group based on the prognostic model constructed from the 21 breast cancer prognostic pathways identified in TCGA. There was a significant difference in survival between these two groups (log-rank test, *p* < 2.2 × 10^−16^, see Figure 8).

### 3.4. Behavior of Prognostic Pathways among Cancer Subtypes

**Figure 9:**
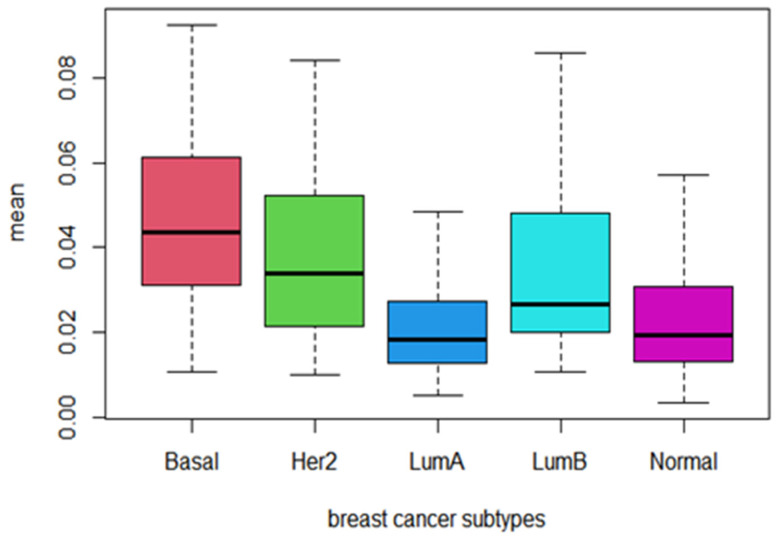
The Boxplot of the mean PDS score of the 21 identified prognostic pathways across Pam50 subtypes of breast cancer.

In addition, we computed the mean values of PDS for the 21 identified prognostic pathways among breast cancer Pam50 subtypes and plotted their distribution in Figure 1. Obviously, the PDS scores of the identified prognostic pathways are higher in Basal-like subtype with poor prognosis than other subtypes, while the PDS sores of subtypes of LumA and Normal-like are relatively lower. As we all know, these two subtypes LumA and Normal-like are usually correlated with low degree of malignancy and good prognosis. This means that the PDS score of the identified prognostic pathways can reflect the degree of malignancy and prognosis of breast cancer among subtypes. The higher the PDS score of the prognostic pathways, the more serious the pathway deregulated and the worse the prognosis (see Figure 9).

### 3.5. Genes in Prognostic Pathways

We examined the annotations of the gene sets of these 21 pathways in breast cancer. The occurrence frequency of genes in all prognostic pathways of breast cancer was statistically analyzed, and the genes with the highest frequency were selected, for example, the mitogen-activated protein kinases *MAPK1*, *MAPK3*, and *MAP2K1*; G-protein-related genes *GNAQ* and *HRAS*; and other oncogenes, such as *RhoA*, *ROCK1*, and *ROCK2*. Increased expression and/or activation of *HRAS* is often associated with tumor aggressiveness in breast cancer. *HRAS* induces the invasion and migration of *MCF10A* human breast epithelial cells, and *HRAS* induces cell proliferation and phenotypic transformation [29]. The *KRAS*, *BRAF*, and *PIK3CA* genes activate the ERK/MAPK pathway [30]. The activation of *NHE1* and subsequent invasion induced by serum deprivation in metastatic human breast cells is coordinated by a sequential *RhoA/p160ROCK/p38* MAPK signaling pathway gated by direct phosphorylation of protein kinase A and inhibition of *RhoA* [31]. In addition, *HRAS*, *KRAS*, *AKT1*, *PIK3CA*, *TP53*, and 24 other genes (see Supplementary Table S4) in these 21 prognostic pathways of BRCA have been proven to be BRCA driver genes, accounting for 28.3% of the total complement of BRCA driver genes [32].

Impressively, for prognosis-related signaling pathways with high correlations, key genes in the MAPK and TGF-β signaling pathways are associated with many cancer types. Through mutation of the pathway members or aberrant activation of the downstream genes (i.e., *RAS*, *SRC*, and *PI3K*) and receptor kinases, the MAPK signaling pathway is overactivated in different malignancies. The activators and components of the MAPK pathway—*Raf*, *RAS*, *BRAF*, *MEK*, and *ERK*—are frequently mutated in colon, melanoma, ovarian, thyroid, colorectal, and non-small cell lung cancers [33]. The TGF-β signaling pathway has multiple gene targets, and TGF-β performs its critical functions in proliferation and suppression by targeting the *c-Myc*, *Cyclin A/B/D/E*, *CDK1/2/4/6*, *p15INK4B*, *p21CIP1*, and *p27KIP1* genes [34].

## 4. Discussion

We applied Pathifier, a recently introduced method for analysis of transcriptomic data, to perform a comprehensive pan-cancer study across 13 different tumor types with more than 6000 cancer samples in TCGA. For each cancer type, the cancer-specific deregulated pathways and prognostic pathways were found to differ greatly among cancers, reflecting the heterogeneity across cancer types. In addition, we constructed a prognostic pathway-based prognostic model, which was well validated in an independent data set in the METABRIC database. The prognostic model accurately distinguished the high-risk and low-risk score groups, indicating the broad applicability of our model as a prognostic model. Then, for any given pair of cancer types, we found that there is little overlap between the two lists of pathway-based biomarkers. These results highlight the observation that cancer is a highly heterogeneous disease and that, therefore, personalized treatment is necessary for patients with different cancer types.

Although we developed an approach for the classification and identification of prognostic pathways in different cancer types, our study has a few limitations. We only used normal samples in TCGA, and a certain number of normal samples are needed to estimate PDS more accurately. In addition, some types of cancer with high heterogeneity can be studied further by subtyping based on pathways.

In summary, Pathifier-based research can allow more accurate and robust identification of prognostic pathways in cancer samples and is expected to improve precision treatment for different cancers. We also expect that our method, with its good performance, will be applicable to other cancers. Future validation in other cancer types with large sample sizes is desired.

## 5. Conclusions

In this study, we analyzed transcriptomic data of 13 different cancer types in TCGA database to identify cancer-specific deregulated pathways and prognostic pathways based on pathway deregulation scores. First, individual-specific pathway deregulation scores for each sample were inferred. Second, the cancer-specific deregulated pathways and prognostic pathways of different cancer types were identified. Finally, we constructed and evaluated the pathway-based prognostic prediction model.

There are indeed several papers building PDS-based Cox models, and all of them are focused on a single type of cancer [35]. However, we performed the pathway based personalized analysis on pan-cancer including 13 types of cancer. The results showed that the individual-specific deregulated pathways score can clearly distinguish different cancer types and their tumor-adjacent tissues. The cancer-specific deregulated pathways and prognostic pathways of different cancer types have high heterogeneity. The cancers with more deregulation pathways tend to be malignant and have worse prognoses. The prognostic model based on pathways successfully predicted survival and prognosis both on training data and validation data. We believe that our prognostic models based on pathways are reliable for prognostic prediction. Most of these results cannot be obtained by PDS-based analysis on only a single cancer type. These are the highlights of our study. In addition, this work may improve the development of precision medicine.

## Figures and Tables

**Figure 3:**
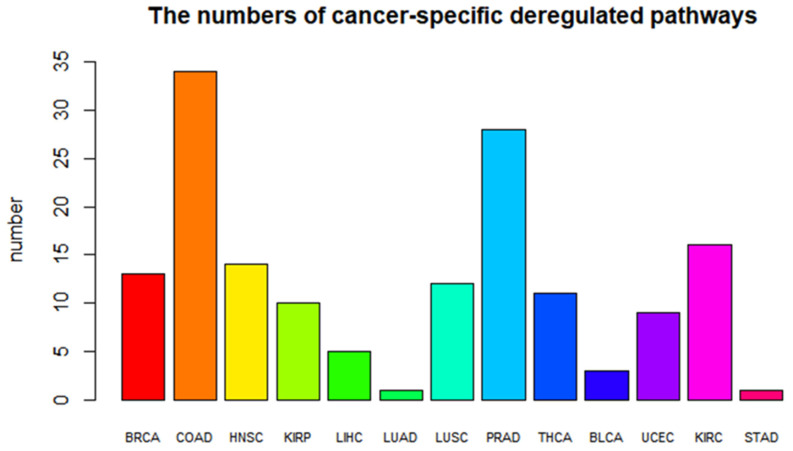
Barplot of the numbers of cancer-specific deregulated pathways. Different colors represent different types of cancer.

**Figure 4:**
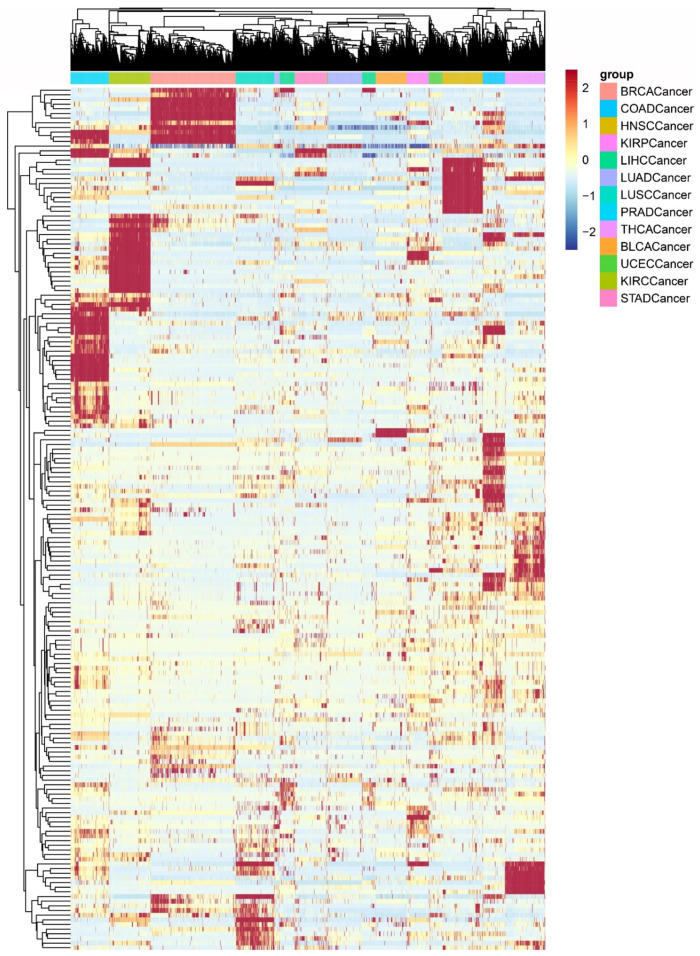
Clustering of PDSs for all cancer samples. The different colors of the bars represent samples of different cancer types. Each row in the matrix represents a pathway; each column represents a sample.

**Table 1:** The numbers of samples of 13 types of cancer downloaded from the TCGA and METABRIC databases.

Cancer Type	TCGA RNA-Seq		METABRIC RNA-Seq
	Tumour Samples	Normal Samples	Tumour Samples
Breast invasive carcinoma(BRCA)	1102	113	1904
Colon adenocarcinoma(COAD)	287	41	
Head and Neck squamous cell carcinoma(HNSC)	522	44	
Kidney renal papillary cell carcinoma(KIRP)	291	32	
Liver hepatocellular carcinoma(LIHC)	374	50	
Lung adenocarcinoma(LUAD)	517	59	
Lung squamous cell carcinoma(LUSC)	502	51	
Prostate adenocarcinoma(PRAD)	498	52	
Thyroid carcinoma(THCA)	513	59	
Bladder Urothelial Carcinoma(BLCA)	408	19	
Uterine Corpus Endometrial Carcinoma(UCEC)	177	24	
Kidney renal clear cell carcinoma(KIRC)	534	72	
Stomach adenocarcinoma(STAD)	415	35	
Total	6140	651	1904

**Table 2:** The performance of the random forest classifier based on PDSs in 13 types of cancer.

Cancer	BRCA	COAD	HNSC	KIRP	LIHC	LUAD	LUSC	PRAD	THCA	BLCA	UCEC	KIRC	STAD
Sn	0.966	0.986	0.972	0.973	0.943	0.987	0.992	0.904	0.973	0.985	0.969	0.968	0.965
Sp	0.998	1	0.991	0.992	0.999	1	1	0.928	0.969	0.82	0.995	0.984	0.999
Acc	0.968	0.988	0.973	0.975	0.949	0.988	0.993	0.906	0.972	0.977	0.971	0.984	0.967

Cancer-specific deregulated pathways were identified by a statistical model, and the number of cancer-specific deregulated pathways varied greatly (see Figure 3). The cancer-specific deregulated pathways of each cancer type are shown in Supplementary Table S1. The numbers of COAD-specific and PRAD-specific pathways are as high as approximately 30, while the numbers of LUAD-specific and STAD-specific pathways are relatively low. The cluster heatmap of the PDS scores of all cancer samples is shown in Figure 4, which also shows cancer-specific deregulated pathways in dark red and dark blue. It is easy to see that samples of the same cancer type are well clustered, that samples of different cancer types are separated from each other, and that cancer-specific deregulated pathways in different cancer types are significantly different.

**Table 3:** The prognostic pathways shared by KIRC and KIRP.

Starch and sucrose metabolism
Riboflavin metabolism
TGF-beta signaling pathway
Prostate cancer
Thyroid cancer
Small cell lung cancer

## Data Availability

RNA-seq data and clinical data for 13 cancer types, including 6140 cancer samples and 651 tumor-adjacent tissues, were downloaded from TCGA database (http://tcga-data.nci.nih.gov/tcga/ accessed on 24 June 2020) by using TCGA-Assembler 2 (ver 2.0.6, http://www.compgenome.org/TCGA-Assembler/). Additional clinical data of all samples were downloaded using the R package RTCGA (version 1.22.0). RNA-seq data and clinical data for 1904 breast cancer samples were downloaded from the METABRIC database (http://www.cbioportal.org/study/summary?id=brca_metabric, accessed on 1 April 2021) for validation, and these data were used as validation data. A total of 185 KEGG pathways were downloaded from MSigDB database (http://www.gsea-msigdb.org/gsea/msigdb/, accessed on 2 March 2020).

## Supplementary Materials

The following are available online at https://www.mdpi.com/article/10.3390/biomedicines9111502/s1, Table S1: The cancer-specific deregulated pathways of each cancer type. Table S2: The prognostic pathways among the different types of cancer. Table S3: The prognostic pathways in the 13 cancers are distributed among different types. Table S4: The driver genes in these 21 prognostic pathways of BRCA.
