# Supplementary material for: Pathway-Based Personalized Analysis of Pan-Cancer Transcriptomic Data
Source: Biomedicines. 2021 Oct 20;9(11):1502. doi: 10.3390/biomedicines9111502 (PMC8615289; doi:10.3390/biomedicines9111502)
Supplement: Supplementary file 1 [file biomedicines-09-01502-s001.zip › Supplementary.pdf]

Supplementary Materials for

"Pathway-based personalized analysis of pan-cancer transcriptomic data"

Overview of Supplementary Materials:

Supplementary Tables

Supplementary Table S1: The cancer-specific deregulated pathways of each cancer type

Supplementary Table S2: The prognostic pathways among the different types of cancer

Supplementary Table S3: The prognostic pathways in the 13 cancers are distributed among different types

Supplementary Table S4: The driver genes in these 21 prognostic pathways of BRCA
